# A randomised controlled multicentre investigator-blinded clinical trial comparing efficacy and safety of surgery versus complex physical decongestive therapy for lipedema (LIPLEG)

**DOI:** 10.1186/s13063-021-05727-2

**Published:** 2021-10-30

**Authors:** Maurizio Podda, Maximilian Kovacs, Martin Hellmich, Rebecca Roth, Marouan Zarrouk, Daria Kraus, Reinhild Prinz-Langenohl, Oliver A. Cornely

**Affiliations:** 1 Department of Dermatology, Medical Center Klinikum Darmstadt, Teaching Hospital Goethe-University Frankfurt, Grafenstr. 9, 64283 Darmstadt, Germany; 2 University of Cologne, Institute of Medical Statistics and Computational Biology, Faculty of Medicine and University Hospital Cologne, Robert-Koch-Str. 10, 50924 Cologne, Germany; 3 Clinical Trials Centre Cologne (ZKS Köln), Faculty of Medicine and University Hospital Cologne, University of Cologne, Gleueler Str. 269, 50935 Cologne, Germany; 4 University of Cologne, Faculty of Medicine and University Hospital Cologne, Department I of Internal Medicine, Excellence Center for Medical Mycology (ECMM), Joseph-Stelzmann-Str. 26, 50931 Cologne, Germany; 5 German Centre for Infection Research (DZIF), Partner Site Bonn-Cologne Department, Herderstr. 52, 50931 Cologne, Germany; 6 Chair Translational Research, Cologne Excellence Cluster on Cellular Stress Responses in Aging-Associated Diseases (CECAD), Faculty of Medicine and University Hospital Cologne, University of Cologne, Joseph-Stelzmann-Str. 26, 50931 Cologne, Germany

**Keywords:** Lipedema, Complex decongestive therapy, Liposuction, Randomisation, Phase 3

## Abstract

**Background:**

Lipedema is a chronic disorder of the adipose tissue that affects mainly women, characterised by symmetrical, excessive fatty tissue on the legs and pain. Standard conservative treatment is long-term comprehensive decongestive therapy (CDT) to alleviate lipedema-related pain and to improve psychosocial well-being, mobility and physical activity. Patients may benefit from surgical removal of abnormally propagated adipose tissue by liposuction. The LIPLEG trial evaluates the efficacy and safety of liposuction compared to standard CDT.

**Methods/design:**

LIPLEG is a randomised controlled multicentre investigator-blinded trial. Women with lipedema (*n*=405) without previous liposuction will be allocated 2:1 to liposuction or CDT. The primary outcome of the trial is leg pain reduction by ≥2 points on a visual analogue scale ranging 0–10 at 12 months on CDT or post-completion of liposuction. Secondary outcomes include changes in leg pain severity, health-related quality of life, depression tendency, haematoma tendency, prevalence of oedema, modification physical therapy scope, body fat percentage, leg circumference and movement restriction.

The primary analysis bases on intention-to-treat. Success proportions are compared using the Mantel-Haenszel test stratified by lipedema stage at a 5% two-sided significance level. If this test is statistically significant, the equality of the response proportions in the separate strata is evaluated by Fisher’s exact test in a hierarchical test strategy.

**Discussion:**

LIPLEG assesses whether surgical treatment of lipedema is safe and effective to reduce pain and other lipedema-related health issues. The findings of this trial have the potential to change the standard of care in lipedema.

**Trial registration:**

ClinicalTrials.gov NCT04272827. Registered on February 14, 2020.

**Trial status:**

Protocol version is 02_0, December 17, 2019

**Supplementary Information:**

The online version contains supplementary material available at 10.1186/s13063-021-05727-2.

## Introduction

### Background

Lipedema was first described in 1940 by Allen and Hines [1] and has a registered diagnosis in the International Classification of Diseases [2] (ICD-10_GM Version 2020) coded stage-specifically (ICD10-E88.20, E88.21, E88.22, and E88.28). This disease, almost exclusively affecting females, is characterised by a symmetrical bilateral increase of the adipose tissue in the upper and/or lower extremities. It is unresponsive to physical activity or dietary intervention [3, 4]. A large proportion of the patients also shows the involvement of the arms [5]. Lipedema is associated with pain of the adipose tissue, increased touch sensitivity, feeling of tension in the affected regions and an increased tendency to haematoma after minor traumas [6]. Data on the prevalence of lipedema is limited. The prevalence of lipedema may be between 10 and 18% among women [7–9]. Child et al. estimated the prevalence within the female population as 1 in 72,000 [3]. However, lipedema is often misdiagnosed as obesity or lymphedema and therefore is incorrectly treated [3, 5]. Shivat et al. have established lipedema diagnostic algorithm to guide clinicians in making the correct diagnosis [10].

The aetiology of lipedema is unknown, but a genetic disposition is likely since lipedema is often seen in patients with a family history of this disease. X-linked dominant or gender-limiting autosomal dominant inheritance is assumed [3]. An important factor in the pathophysiology of lipedema is the increased capillary permeability, which leads to orthostatic edema and seems to be responsible for the increased tissue sensitivity in response to touch and pressure. Increased capillary fragility could explain the tendency towards haematoma formation [11].

The course of the disease is individual: some patients develop lipedema at a lower degree of severity and with stabilisation. Other patients show progression after stressful situations like pregnancy or surgery [12]. The onset of lipedema is common in teenage years, but is also observed after pregnancy or even menopause [3, 13].

Lipedema is divided into three stages according to skin morphology and palpation: stage I is characterised by smooth skin surface, thickened subcutis and small-nosed fat structure. In stage II, nodules appear in the fatty tissue and the skin surface is uneven, while in stage III, the tissue is hardened and rough with the presence of large deforming fat flaps [4].

The comprehensive decongestive therapy (CDT) is part of the standard conservative treatment [6]. Originally developed for the treatment of lymphedema, improvement has also been seen in patients with lipedema (reduction in leg circumference, tension, and pressure pain). As the treatment success is short-term, therapy is permanently necessary [4, 12].

CDT consists of a combination of lymphatic drainage, compression therapy (usually flat-knitted compression stockings), skin care and physical activity. It is unclear whether CDT influences the progression of pain in addition to the abovementioned benefits. However, the specific fat accumulation and distribution with the accompanying pain and tendency to form haematomas remain [5]. Physical activity, if necessary in combination with a dietary intervention, aims to reduce or prevent obesity, which has a negative prognostic influence on the development of lipedema [6, 10].

Starting in the 1990s, liposuction has emerged as the most important surgical intervention for lipedema. The pathologically altered subcutaneous fatty tissue is surgically removed without damaging the lymphatic system. Liposuction methods are tumescent anaesthesia (TA) and water-assisted liposuction (WAL). In TA, several litres of an anaesthetic solution are infused into the subcutaneous area (‘wet technique’) so that the fat cells swell and vessels constrict. Then, the subcutaneous fat is suctioned off by a very fine, blunt, vibrating cannula (power/vibration-assisted liposuction, PAL). In WAL, a jet of water releases the fat cells from the connective tissue and a mixture of fat cells, water and tumescent solution is aspirated [14–16]. This method is a tissue-friendly surgical method and minimises the risk of injuring vascular, lymphatic and nerve structures [4, 14, 17–19]. TA is standard for surgical treatment nowadays and recommended as the method of choice in the guidelines for lipedema [6].

Liposuction leads to reduction of lipedema-specific fat accumulation and distribution, pain sensitivity, edema, tendency to haematoma and restriction of movement [4, 19]. An improvement in the quality of life and a decrease in the need for conservative therapy has been shown [4, 11, 20–22].

Liposuction is therefore discussed as a useful addition to or even alternative therapy for CDT, especially in the early stages of lipedema in order to prevent possible long-term effects such as the development of secondary lymphedema and disease progression, to improve the quality of life and to reduce the need for CDT [4, 11, 20–22].

In summary, clinical experiences as well as small trials demonstrate a positive effect of liposuction in lipedema with respect to pain, movement improvement and quality of life. However, controlled and randomised clinical trials comparing conservative and surgical treatments with valid statistical methods are missing. There is no evidence that surgical treatment is equivalent or even superior to conservative treatment with respect to pain in lipedema stages I, II and III. Therefore, a large-scale randomised controlled investigator-blinded trial will be performed with the aim to provide a definitive answer.

### Rationale

Complex decongestive therapy is the standard of care for patients with lipedema. Liposuction is a non-standard alternative. The rationale of the LIPLEG trial is to compare effectiveness using leg pain reduction as the primary outcome in the two treatment groups.

### Objectives

The primary objective of the trial is to investigate whether the surgical treatment (treatment A) is superior to conservative treatment (treatment B) by comparing leg pain after 12 months. The secondary objective is to estimate the potential benefit of liposuction for the patient by evaluating changes in the severity of pain in the legs, health-related quality of life, depression and haematoma tendency, prevalence of edema, modification in the scope of conservative therapy, body fat percentage, leg circumference and movement restriction.

#### Primary outcome

The primary outcome is the reduction of pain in the legs 12 months after completion of liposuction or after 12 months of treatment by CDT upon randomisation, respectively (≥ 2 points on a numerical rating scale with range 0–10; German pain questionnaire).

#### Secondary outcomes

1. Change in the severity of the pain in the legs
2. Change in health-related quality of life according to Short Form 36 (SF-36), Dermatology Life Quality Index (DLQI) and World Health Organization Quality of Life-abbreviated version of the WHOQOL 10 (WHOQOL-BREF)
3. Altered depression tendency according to Patient Health Questionnaire-9 (PHQ-9)
4. Altered haematoma tendency
5. Prevalence of edema
6. Modification in the scope of physical therapy
7. Body fat percentage
8. Change of leg circumference
9. Reduction of the movement restriction (Lower Extremity Functional Scale, LEFS cumulative score)
10. Occurrence of (serious) adverse events during the trial

### Trial design

LIPLEG is a multicentre, controlled, randomised, investigator-blinded trial. Female patients with lipedema interested in participating in the trial could register in an online portal until the end of December 2019. The patients are randomly drawn and assigned to a trial site according to the nearest post code. Then, the patients will be contacted by the trial sites and assessed for eligibility by telephone interview. If the patient is assessed as eligible, a screening visit on-site will take place in order to check the inclusion and exclusion criteria. Patients will be considered for enrolment when all inclusion criteria are confirmed, and none of the exclusion criteria is fulfilled. Four hundred five patients are planned to be included in the trial and assigned in a ratio of 2:1 to treatment group A (liposuction) or B (CDT). The investigator assessing the primary and secondary outcomes will be blinded. The surgeon, patients and further investigators will not be blinded for the allocated treatment. Since lipedema mainly affects females, males will not be included in the study.

## Methods

### Participants, interventions and outcomes

#### Study setting

The trial will be performed in > 10 trial sites in Germany, which are listed on ClinicalTrials.gov NCT04272827.

#### Trial sites

Only specialised trial sites (Dermatology or Plastic Surgery) are eligible for participation in the trial. Only principal investigators (PI) and participating trial sites that meet the necessary requirements, with qualifications and experience to conduct a clinical trial, are selected for the trial. Furthermore, only surgeons with sufficient experience in liposuction surgery can perform the liposuction in the trial. The sponsor designates the PI at each trial site, who selects qualified and experienced personnel to conduct the trial.

All trial sites will be trained during the initiation visit by the clinical research associate (CRA).

##### Eligibility criteria

*Inclusion criteria*

1. Signed informed consent form
2. Female
3. Age ≥18 years
4. Lipedema of the legs stages I, II or III
5. Average pain in the legs over the last 4 weeks ≥ 4 points on a numerical rating scale
6. Documentation of an insufficient complaint relief by conservative methods
7. Willingness and ability to take conservative measures according to the study plan, during and, if necessary, after the liposuction treatment
8. Full legal capacity

*Exclusion criteria*

1. Simultaneous lipedema of the arms and legs in which the involvement of the arms influences the primary outcome
2. Previous liposuctions
3. Diseases affecting operability (e.g. cardiac diseases, coagulation disorders, metabolic diseases, body weight >120kg, active infectious diseases, epilepsy, diseases requiring immunosuppression or anticoagulation, allergies to drugs used before and during surgery)
4. Diseases influencing adequate CDT, e.g. heart failure (volume stress), lack of physical ability to wear compression stockings (e.g. joint disease, neurological deficits).
5. Primary obesity without disproportion and without reference to lipedema
6. Secondary obesity
7. Fat distribution disorders of other genesis (e.g. painless lipo-hypertrophy, benign symmetrical lipomatosis or lipomatosis dolorosa)
8. Other edema causing diseases (e.g. lymphedema, phlebedema or myxedema)
9. Lack of willingness to provide adequate contraception
10. Positive pregnancy test
11. Lactation period
12. Usage of a lymphomat
13. Participation in other clinical trials
14. Cosmetic motivation for participation in the trial

#### Interventions

In order to establish same starting conditions for all patients before randomisation, the trial participants initially receive CDT as recommended by the investigator in a run-in phase. The first part (phase I) of the run-in phase serves to eliminate edema within a maximum period of 4 weeks, followed by the second part (phase II), in which patients are treated with standardised CDT for a further 6 months to maintain the results achieved in phase I. Patients are then randomly assigned to one of the two treatment groups (treatment A: liposuction, treatment B: CDT) provided that all inclusion criteria are still met (and no exclusion criterion is fulfilled).

In the liposuction group, patients receive liposuction treatment using the wet technique. Techniques such as vibration (PAL) or water jet (WAL) can support the procedure depending on the amount of fat that is to be removed. A maximum of four surgical sessions is possible. CDT therapy is continued to the necessary extent for progression prophylaxis.

The CDT group is treated with CDT alone for further 12 months upon randomisation to maintain at least the results obtained at the end of the run-in phase. After this period, patients will be offered liposuction treatment provided all inclusion and exclusion criteria are still valid.

##### Study visits and assessments

**Fig. 1:**
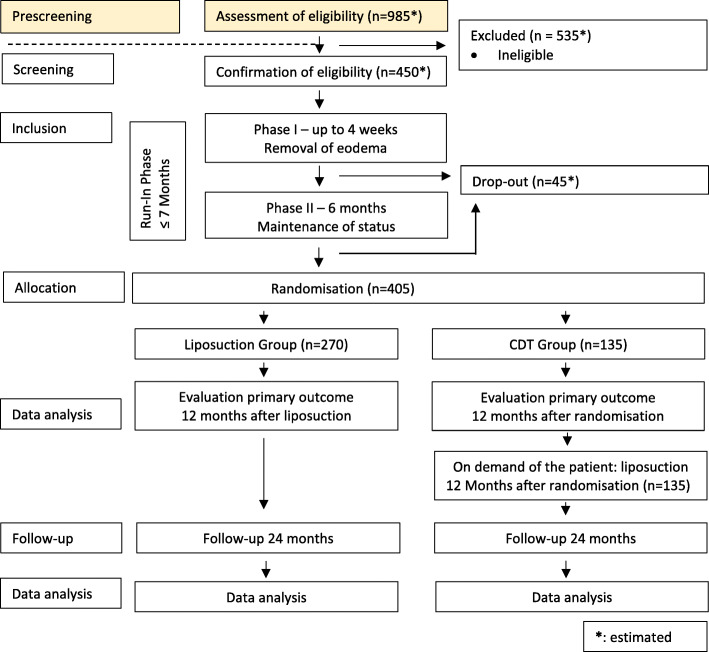
Trial flowchart. Abbreviations: *CDT* comprehensive decongestive therapy

A screening visit (baseline visit) on site will be performed by the investigator including written informed consent, check of inclusion and exclusion criteria, and medical history. The following parameters are recorded on the screening visit as well as on the subsequent visits: weight, supplementary medications, leg circumference, occurrence of leg edema, haematoma tendency, validated questionnaires for the determination of pain (German pain questionnaire), quality of life (SF-36, DLQI, WHOQOL-BREF), depression (PHQ-9), overall impairment modified according to Schmeller [23] and functional limitations (LEFS). All patients will then receive CDT during a run-in phase of maximal 7 months. After randomisation, the patients receive either liposuction and CDT in the following observation period of 36 months or CDT alone. After 12 months of observation, patients of the CDT group can opt for liposuction surgery followed by another 24 months of observation. During the trial, physical activities are permitted as an optional adjuvant treatment for lipedema. The supportive use of a lymphomat is not permitted during the trial. Compliance of patients with the trial procedures will be monitored by questionnaires and clinical examination during each study visit. Visits and assessments are described in more detail in Tables 1 (liposuction group) and 2 (CDT group). A detailed trial flowchart is shown in Fig. 1.

**Table 1:** Visit schedule—liposuction group

Liposuction group		Run-in phase				12 months of observation		24 months of follow-up			
Visit	Screening *Day 0*	P1 *≤4 wks*	P2 *6 mo. after P1*		Lipo-suction(s) *1–4 mo. after P2*	V1 *6 mo.*	V2 *12 mo.*	V3 *6 mo.*	V4 *12 mo.*	V5 *18 mo.*	V6 *24 mo.*
						*After completion of liposuctions(s)*		*After V2*			
Informed consent	x			**Liposuction of the legs (2–4 surgeries at intervals of 5–7 weeks)**							
Information about liposuction	x				x						
In-/exclusion criteria	x		x								
Demographic data	x										
Anamnesis	x										
Concomitant medication	x	x	x		x	x	x	x	x	x	x
Weight	x	x	x			x	x	x	x	x	x
Leg circumference	x	x	x			x	x	x	x	x	x
Leg oedema	x	x	x			x	x	x	x	x	x
Body fat	x	x	x			x	x	x	x	x	x
Leg volume (optional)	x	x	x			x	x	x	x	x	x
Arm circumference	x	x	x			x	x	x	x	x	x
Skin appearance	x	x	x			x	x	x	x	x	x
Quality of life (SF-36, DLQI, WHOQOL-BREF)	x	x	x			x	x	x	x	x	x
Depression (PHQ-9)	x	x	x			x	x	x	x	x	x
Hematoma (mod. to Schmeller et al. 2010)	x	x	x			x	x	x	x	x	x
Overall impairment (mod. to Schmeller *et al*. 2010)	x	x	x			x	x	x	x	x	x
Pain (German Pain questionnaire)	x	x	x			x	x	x	x	x	x
Functional limitations (LEFS)	x	x	x			x	x	x	x	x	x
Randomisation			x								
ECG					x						
Laboratory data					x						
Pregnancy test	X		x		x						
Adverse events		x	x		x	x	x	x	x	x	x
Liposuction					x						
Query on the necessity of recurrent liposuction						x	x	x	x	x	x
Query on the scope of CDT	x	x	x		x	x	x	x	x	x	x

Abbreviations: *wks* weeks, *mo* months, *SF-36* Short Form 36, *DLQI* Dermatology Life Quality Index, *WHOQOL-BREF* World Health Organization Quality of Life-abbreviated version of the WHOQOL 100, *PHQ-9* Patient Health Questionnaire-9, *LEFS* Lower Extremity Functional Scale, *CDT* comprehensive decongestive therapy

**Table 2:** Visit schedule—CDT group

CDT Group		Run-in phase		12 months of observation				24 months of follow-up			
Visit	Screening *Day 0*	P1 *≤4 wks.*	P2 *6 mo. after P1*	V1 *6 mo.*	V2 *12 mo.*		optional: Lipo-suction(s) 1-4 *mo. after V2*	V3 *6 mo.*	V4 *12 mo.*	V5 *18 mo.*	V6 *24 mo.*
				*After randomisation*				*After completion of liposuction(s) or V2*			
Informed consent	x					**On demand of the patient: liposuction of the legs (2–4 surgeries at intervals of 5–7 weeks)**					
Information about OP	x						x				
In-/exclusion criteria	x		x		x						
Demographic data	x										
Anamnesis	x										
Weight	x	x	x	x	x			x	x	x	x
Concomitant medication	x	x	x	x	x		x	x	x	x	x
Leg circumference	x	x	x	x	x			x	x	x	x
Leg oedema	x	x	x	x	x			x	x	x	x
Body fat	x	x	x	x	x			x	x	x	x
Leg volume (optional)	x	x	x	x	x			x	x	x	x
Arm circumference	x	x	x	x	x			x	x	x	x
Skin appearance°	x	x	x	x	x			x	x	x	x
Quality of life (SF-36, DLQI, WHOQOL-BREF)	x	x	x	x	x			x	x	x	x
Depression (PHQ-9)	x	x	x	x	x			x	x	x	x
Hematoma (mod. to Schmeller et al. 2010)	x	x	x	x	x			x	x	x	x
Overall impairment (mod. to Schmeller et al. 2010)	x	x	x	x	x			x	x	x	x
Pain (German Pain questionnaire)	x	x	x	x	x			x	x	x	x
Functional limitations (LEFS)	x	x	x	x	x			x	x	x	x
Randomisation			x								
ECG							x				
Laboratory data							x				
Pregnancy test	X				x		x				
Adverse events		x	x	x	x		x	x	x	x	x
Liposuction							x				
Query on the necessity of recurrent liposuction								x	x	x	x
Query on the scope of CDT	x	x	x	x	x		x	x	x	x	x

Abbreviations: *wks* weeks, *mo* months, *SF-36* Short Form 36, *DLQI* Dermatology Life Quality Index, *WHOQOL-BREF* World Health Organization Quality of Life-abbreviated version of the WHOQOL 100, *PHQ-9* Patient Health Questionnaire-9, *LEFS* Lower Extremity Functional Scale, *CDT* comprehensive decongestive therapy

##### Ancillary and post-trial care

For the optional ancillary treatment of lipedema, physical activities, especially water sports, are permitted, as recommended in the guidelines. However, the type and intensity of these should not be changed for the duration of the study. The supportive use of lymphomatics, on the other hand, is not permitted in the context of a desired homogeneous group of patients. After the end of the clinical trial, participants will be treated according to standard of care.

#### Withdrawal

Patients can withdraw at any time during the trial without providing a reason and without any personal disadvantage. A patient may also be withdrawn if, on the basis of the investigator’s judgement, continuation of the trial may be detrimental to the participant’s health. Further reasons for the investigator to discontinue a patient’s trial participation includes, e.g. insufficient compliance, patient becomes pregnant before reaching the primary outcome, inclusion and exclusion criteria are no longer met after the run-in phase. Reasons for all withdrawals will be recorded in the patient’s medical file and their electronic case report forms (eCRFs). All data will be analysed according to the intention-to-treat principle.

### Outcomes

#### Outcome measures and efficacy assessment

Efficacy assessments include the primary and secondary outcomes described above. The choice of these measuring instruments is based on the fact that lipedema has far-reaching effects on various socially relevant areas of the patient’s life that are covered by these measuring instruments.

Data will be recorded on eCRF. The primary outcome, pain in the legs, is collected either 12 months after the final liposuction surgery in the liposuction group or 12 months after randomisation in the CDT group by using the German Pain Questionnaire [24]. Occurrence of adverse events (AEs) and serious adverse event (SAEs) during the trial will be documented in the eCRF.

### Participant timeline

#### Proposed overall timescale

(see Supplementary Material)

### Sample size

#### Determination of trial size

According to the results of Schmeller et al. [22, 23], the success proportion of the intervention at each stage is expected to be at least 40% and that of the control to be at most 10%. The chi-square test requires 111 patients (74 vs. 37) per group to reach 80% power at two-sided significance level of 5% to detect such a difference. To compensate for the influence of dropouts and stratification, 135 patients per stage are included (this corresponds to an increase of approx. 20%). Accounting for up to 10% attrition from baseline to follow-up, the number of 450 patients is required in total.

### Assignment of interventions

#### Patient randomisation

Patients are randomly assigned to the treatment groups (liposuction or CDT alone) centrally using the 24/7 Internet randomisation service ALEA (FormsVision BV, Abcoude, NL) in a 2:1 ratio (liposuction: CDT alone) based on permuted blocks of varying length as provided by ALEA using a pseudo-random number generator. Assignment is stratified by stage of lipedema and trial site. The randomisation service is set up and maintained by the Institute of Medical Statistics and Computational Biology, University of Cologne. Programming (using a web-interface) and validation of the service are done according to the Institute’s SOPs. The assignment of the groups can be made by all investigators, except the investigator (blinded investigator) carrying out the clinical assessments and questionnaires after surgery who should not be aware of who is receiving liposuction or CDT. The randomisation sequence is concealed from all clinical staff, i.e. only ALEA service administrators have access to the sequence.

#### Blinding (masking)

This study is a single-blind (investigator-blinded) trial. After randomisation, all examinations will be conducted by an investigator who will be blinded to the treatment allocation. Therefore, the surgical scars are taped by unblinded study personnel before each visit (dummy patches in the control group) and patients are asked not to disclose the received therapy to the blinded investigator. The blinded examination of the patient must not be performed by a surgeon or an unblinded investigator. The blinded investigator is not allowed to access the database and is informed that he is not allowed to view the patient’s file of the patient he is examining (except in an emergency). The data collected by the blinded investigator are documented on working sheets and entered into the eCRF by unblinded study personnel in a timely manner.

The analysing statistician will also be kept masked to treatment assignment until the final statistical analysis is completed.

### Data collection, management, and analyses

#### Data collection and management

The IT infrastructure and data management are provided by the Clinical Trials Centre Cologne (CTCC).

The trial database is developed and validated by CTCC on the basis of standard operating procedures. All changes made to the data will be documented in an audit trail. The commercial online software TrialMaster^TM^ (Anjusoftware.com) is used as a data management system, ensuring data safety by a firewall and backup system and including multiple data storage sites. The data are backed up daily.

All relevant trial data collected during the trial are documented soon after data gaining and entered into the eCRF by the responsible investigators or a person authorised by the principal investigator (exception of the blinded investigator).

This includes all outcomes measures and questionnaires. Automatic plausibility checks are run during data entry to immediately detect discrepancies. Data management of the CTCC carries out checks on the completeness and plausibility of the study data and clarifies all queries with the study centre electronically via the trial software. These queries must be answered promptly by the study centre. The eCRFs are signed by the principal investigator at the end of a patient’s trial participation to confirm the accuracy of the data. After completion and cleaning of the data, the database is closed and the data will be exported for statistical analysis.

To monitor risks and ensure the data quality of the trial, a central quality control process (CQC) is set up.

Using CQC, the performance of the trial sites indicating the documentation rate, documentation quality, and recruitment rate will be evaluated. In the case of conspicuous data (e.g. incorrect or insufficient documentation), this quality control process allows for early intervention and correction of errors.

#### Data protection

The provisions of the EU data protection basic regulation and data protection laws including their national implementation in Germany are observed. It is ensured that all investigational materials and data are pseudonymised in accordance with data protection regulations before scientific processing.

Patients will be informed that their pseudonymised data will be passed on in accordance with provisions for documentation and notification pursuant to § 12 and § 13 of the Good Clinical Practice (GCP) regulations to the recipients described there. Patients, who do not agree to the data handling as described in the informed consent, will not be included in the trial.

### Statistical methods

The primary analysis is according to the intention-to-treat. The primary outcome ‘success’ is evaluated by the stratified (by stage) Mantel-Haenszel test [25] at two-sided significance level of 5% (H_0_: odds ratio = 1 vs. H_A_: odds ratio ≠ 1). If this test is statistically significant, the equality of the success rates in the separate stages is evaluated by Fisher’s exact test (hierarchical test strategy). A missing primary outcome or change of the randomly assigned treatment is counted as failure. No interim analysis is planned. Secondary is the analysis of patients treated and observed per protocol (i.e. completed liposuction or ≥80% of planned CDT sessions done; valid primary outcome). Secondary and further outcomes are evaluated without adjusting for multiple hypothesis testing (i.e. no alpha correction is applied). Distributions of quantitative variables are described as mean (standard deviation) and percentiles (0, 25, 50, 75, 100), qualitative variables are summarised by count and percentage. The impact of missing outcome data is assessed by sensitivity analyses based on single/multiple imputation. The information on AEs is assessed (according to severity, intensity of symptoms, causal relationship to the CDT or intervention), coded and summarised by contingency tables. Subgroup analysis is done by patient age, study centre and surgeon.

### Monitoring

The trial sites will be monitored closely to ensure patient’s safety and the quality of the data collected. Monitoring is performed risk-based. The CRA will review informed consent forms, verify the completeness and accuracy of the patient data collected and ensure that the trial is conducted in accordance with the protocol, ICH-GCP principles and legal requirements.

Each trial site received a site selection visit to ensure staff qualification and experience, and sufficient capacity and equipment. The initial training of each site was performed during an initiation visit prior to trial start. A total of 140 regular monitoring visits are planned. The frequency of the regular monitoring visits at the trial site depends on the recruitment rate and the quality of the documented data at the trial site. Each site will also receive a close-out visit, for formal termination of the trial at the respective trial site.

#### Data Monitoring Committee

A Data Monitoring Committee (DMC) oversees the safety of the patients in the trial by periodically assessing selected trial safety datasets and their influence on the risk-benefit assessment for patients and the justifiability of further trial conduct. The DMC receives a list of AEs and SAEs at regular intervals. As described in a separate DMC manual, the assessment of SAEs is based on the absolute rates, and the difference in rates and the temporal development of the SAEs that occurred. Based on this, the DMC will make recommendations on continuation, modifications or even termination of the trial to the sponsor. It consists of two physicians and a statistician, who are independent experts, not otherwise involved in the conduct of the trial, and operate based on an agreed charter.

#### Adverse events and safety reporting

All AEs and SAEs occurring during the trial will be documented in the patient’s medical records and the eCRF, including date and time of onset and resolution, severity, causal relationship with CDT/liposuction, seriousness and measures. In addition, SAEs are immediately sent to the principal coordinating investigator (PCI) via an automatic e-mail notification directly from the database. The risk-benefit ratio will be checked by the PCI based on a list of AEs that have occurred up to that point, which is made available at regular intervals by the CTCC. All safety-relevant events will be promptly reported to the Ethics Committees and the DMC.

#### Auditing

No auditing is planned during the trial. However, the sponsor has the right to carry out quality assurance audits at the trial site and other institutions that may be involved in the study, whenever deemed necessary. The aim of audits is to check the validity, verifiability and completeness of the data and the credibility of the clinical study as well as to check whether patients’ rights and safety are guaranteed. The sponsor can commission people who are otherwise not involved in the clinical trial (auditors). They are permitted to inspect all trial-related documents (in particular: trial protocol, questionnaires, patient files, trial-related correspondence).

The sponsor and all participating trial sites commit to support auditors and, in this context, to grant the authorised persons access to the original documents.

All auditors commit to treat personal and other data confidentially.

### Ethics and dissemination

The trial was approved by the following Ethics Committees: Ethics Committee of the Hesse State Chambers of Physicians (corresponding Ethics Committee; reference number Az. 2019-1377-evBO), Ethics Committee of the Medical Association of North Rhine (reference number: 2019316), the Ethics Committee of the Saxon State Chambers of Physicians (reference number: EK-BR-76/19-1), Ethics Committee of the Brandenburg State Chambers of Physicians (reference number: AS 117(bB)/2019), Medical Ethics Committee of the University of Oldenburg (reference number: 2020-009), and Ethics Committee of the Baden-Württemberg State Chambers of Physicians (reference number: B-F-2019-075). The ethics committee of the University of Regensburg and of Bavarian Medical Association does not require an additional ethics vote, after the study protocol has been approved by other German ethics boards. In case of important protocol modifications, the PCI will inform the PIs of all trial sites. They will then forward the amendments to the associated ethics committees and trial participants. Written informed consent is provided by all participants prior to any trial procedure. A patient insurance policy is taken out for all included patients. Address, insurance number, telephone and fax number of the insurance company are included in the informed consent. In addition, patients will be insured for travel to the way to the regarding trial site.

The trial is conducted in accordance with the International Conference on Harmonisation for Good Clinical Practice (ICH-GCP). The trial will always comply with the Declaration of Helsinki.

The results of this trial will be submitted for publication in peer-reviewed scientific journals and presented at national and international conferences.

## Discussion

Lipedema, first described by Allen and Hines in 1940 [1], is a disease of adipose tissue that appears almost exclusively in women, mostly at a time of hormonal change (e.g. puberty, pregnancy or menopause). It is believed to be a genetic disease, probably with an X-linked dominant inheritance, or autosomal dominant with sex limitations [3].

The phenotype is characterised by a typical bilateral disproportionate distribution of adipose tissue on the extremities. Main symptoms include a progressive symmetrical non-pitting, fatty swelling of the extremities, with recessing of the hands/feet, easy bruising, pain and lack of response to weight reducing diets [1].

Up to now, there are no curative therapies for lipedema. As an insufficiently managed lipedema will likely progress to lipo-lymphedema, the management of the disease is crucial and consists of two main goals: firstly, improvement of the subjective discomforts (e.g. pain) and secondly, the prevention of further complications (lipo-lymphedema, skin infections, psychological morbidities and mechanically caused complaints such as disorder of gait and joint deformities) [26].

Conservative treatment such as manual lymph drainage, multi-layered short stretch bandaging or medical compression stockings can ameliorate the pain of lipedema patients and prevent a concomitant edema formation [27]. A reduction of the pathological adipose tissue with conservative treatment is not possible [26].

Liposuction as a surgical intervention is an accepted therapeutic approach for the treatment of lipedema, especially when conservative therapies are insufficient or have failed [26, 28]. Liposuction is used for a reduction of the pathological subcutaneous fat tissue and aims to improve the mobility and subjective complaints by reducing the fat tissue volume of the affected extremity.

Up to now, conclusive long-term data are still missing, but short-term results seem promising, especially when the intervention is performed in an early stage of the disease in wet-technique with blunt cannulas [11, 22, 29].

The LIPLEG trial assesses whether surgical treatment of lipedema is safe and effective to reduce pain and other lipedema-related health problems.

Moreover, this study is the first randomised controlled and blinded study to compare conservative to surgical treatment. The findings of this trial will potentially influence standard of care in lipedema.

### Trial status

The authors confirm that the trial was registered prior to the submission of this manuscript (ClinicalTrials.gov Identifier: NCT04272827, registered on 14 February 2020). Recruitment of participants is expected in December 2020, last patient’s last visit is expected in summer 2025.

## Supplementary Information

**Additional file 1. ***SPIRIT 2013 Checklist*

## Data Availability

Not applicable

## Abbreviations

AE: Adverse event
CDT: Comprehensive decongestive therapy
CQC: Central quality control
CRA: Clinical research associate
CTCC: Clinical Trials Centre Cologne
DLQI: Dermatology Life Quality Index
DMC: Data Monitoring Committee
EC: Ethics committee
eCRF: Electronic case report form
IMSB: Institute of Medical Statistics and Computational Biology
LEFS: Lower Extremity Functional Scale
PCI: Principal Coordinating Investigator
PHQ-9: Patient Health Questionnaire-9
PI: Principal Investigator
SAE: Serious adverse event
SF-36: Short Form 36
TA: Tumescent anaesthesia
WAL: Water-assisted liposuction
WHOQOL-BREF: World Health Organization Quality of Life-abbreviated version of the WHOQOL 10
